# Effect of crocin and naringenin supplementation in cryopreservation medium on post-thaw rooster sperm quality and expression of apoptosis associated genes

**DOI:** 10.1371/journal.pone.0241105

**Published:** 2020-10-29

**Authors:** Mahdieh Mehdipour, Hossein Daghigh Kia, Abouzar Najafi, Hossein Mohammadi, Manuel Álvarez-Rodriguez

**Affiliations:** 1 Department of Animal Science, College of Agriculture, University of Tabriz, Tabriz, Iran; 2 Department of Animal and Poultry Science, College of Aburaihan, University of Tehran, Tehran, Iran; 3 Department of Biomedical & Clinical Sciences (BKV), BKH/Obstetrics & Gynaecology, Faculty of Medicine and Health Sciences, Linköping University, Linköping, Sweden; Friedrich-Loeffler-Institute, GERMANY

## Abstract

The aim of our study was to examine the effects of crocin (0.5 (C0.5), 1 (C1) and 1.5 (C1.5) mM) and naringenin (50 (N50), 100 (N100) and 150 (N150) μM) in cryopreservation extender for freezing rooster semen. Sperm motility, viability, abnormalities, membrane functionality, active mitochondria, apoptosis status, lipid peroxidation (LP), GPX, SOD, TAC, the mRNA expression of pro-apoptotic (CASPASE 3) and anti-apoptotic (Bcl-2) genes, fertile eggs, hatched eggs and hatching rate were investigated following freeze-thawing. C1 and N100 resulted in higher (P < 0.05) total motility and progressive motility in comparison to the control group. The C1 and N100 groups improved viability, membrane functionality and reduced lipid peroxidation. We found higher values for active mitochondria with C1 and N100 compared to control group. The C1 and N100 groups showed lower percentages of early apoptosis when compared with control group. Also, C1 and N100 had higher TAC, compared to the control group. The mRNA expressions of BCL-2 in the C1 and N100 groups were significantly higher than that of other treatments. The expression of CASPASES 3 was significantly reduced in C1 and N100 group (P < 0.05) when compared to control group. Significantly higher percentages of fertile eggs, hatched eggs and hatching rate were observed in C1 and N100 compared to the control group. In conclusion, crocin at 1 mM and naringenin at 100 μM seem to improve the post-thawing rooster semen quality, fertility and could protect the sperm by reducing the pro-apoptotic (CASPASE 3) and increasing anti-apoptotic (Bcl-2) genes.

## 1. Introduction

Despite its utilization over 70 years ago [1], cryopreservation of poultry sperm leads to low fertility, which limits its application in genetic stock preservation [2]. Cryopreservation causes harmful effect on sperm which decreases sperm viability and motility [3]. Avian sperm are particularly susceptible to oxidative stress [4], though reactive oxygen species (ROS), in physiological quantities, are necessary for important sperm events leading to successful fertilization [5]. Oxidative stress disturbs motility and mitochondrial activity in sperm, also induces lipid peroxidation of the membrane [6], and DNA fragmentation [7].

Adding antioxidant compounds to the freezing medium is known as one of the ways to reduce the harmful effects of excessive ROS on sperm fertility after thawing because they block or inhibit oxidative stress. Antioxidants provide positive effects on semen and improve sperm parameters such as motility and membrane integrity [8–10].

Crocin is a glycosyl ester of crocetin (one of the carotenoids extracted from saffron) [11]. In an experiment that was performed under in vitro conditions, crocin improved deer sperm motility [12]. This antioxidant can influence sperm physiology through its protective effect on sperm cryopreservation extender.

Naringenin is known as a natural flavonoid that has been studied for some of the most prominent properties containing antioxidant, antiproliferative, anti-inflammatory, and antimutagenic ones [13]. It was observed in previous studies that naringenin protects the cells from arsenic-induced oxidative damage [14, 15].

To the best of our knowledge, no similar study has been performed to evaluate the potential effect of naringenin and crocin in cryopreservation of rooster semen. The objective of this study was to determine the effect of different levels of crocin and naringenin in the extender on cryopreservation of rooster sperm quality after thawing and expression of apoptosis associated genes. The fertility analyses of the post-thaw semen was also performed after the freezing and thawing process.

## 2. Materials and methods

### 2.1. Chemicals and ethics

All chemicals used in this experiment were purchased from Sigma (St. Louis, MO, USA) and Merck (Darmstadt, Germany) chemical companies. Approval for the present experiment was given by The Research Ethics Committees of the University of Tabriz.

### 2.2. Rooster and semen collection

This study was performed on ten adult Ross 308 broiler breeder roosters) 30 weeks old) which were kept individually in cages (diet compositions included: 12% crude protein and 2,750 kcal maintenance energy/kg). The experiment was performed in five replicates on 5 collection days with a frequency of twice per week. Ejaculates were collected using the dorso-abdominal massage method and collection was always performed by the same person and under the same condition [16]. Semen samples from each rooster were analyzed individually. The samples that had the standard criteria motility of >80%, concentration of >3 × 10^9^ sperm/mL (sperm concentration was determined using a hemocytometer (HBG, Berlin, Germany)) and volume of >0.2 mL were used in the study. Over all replicates, 2 ejaculates (of all 50 ejaculates) were rejected. The pooled semen samples were then split according to the number of treatments (seven equal aliquots).

### 2.3. Extender preparation and cryopreservation

Seven experimental groups were applied in this study for semen dilution and diluted (1:20; v/v) at 37°C with Beltsville extender (Table 1): Beltsville extender [17] without antioxidant (control), C0.5 (Beltsville extender with 0.5 mM crocin), C1 (Beltsville extender with 1 mM crocin), C1.5 (Beltsville extender with 1.5 mM crocin), N50 (Beltsville extender with 50 μM naringenin), N100 (Beltsville extender with 100 μM naringenin), N150 (Beltsville extender with 150 μM naringenin). Glycerol was added to the extender at 3.8% (v/v). Next, diluted semen samples were aspirated into 0.25 ml French straws (IMV, L’Aigle, France) to obtain the concentration of 100 × 10^6^ sperm/mL. Consequently, the straws were sealed by polyvinyl alcohol powder and equilibrated for 3 h at 4°C. Then, after equilibration time, the straws were cryopreserved in liquid nitrogen (LN) vapor (4 cm above the LN for 7 min in a cryobox) [18]. Finally, the straws were plunged into LN for storage until thawed and used for assessment of sperm parameters. The frozen straws were thawed individually at 37°C for 30 s in a water bath, and then they were evaluated individually. Before any evaluation, glycerol was removed using a discontinuous Accudenz gradient [19].

**Table 1:** Composition of the Beltsville extender.

Ingredients	
Potassium citrate tribasic monohydrate (g)	0.64
Sodium-L-glutamate (g)	8.67
Magnesium chloride anhydrous (g)	0.34
D-(−)-Fructose (g)	5
Potassium phosphate dibasic trihydrate (g)	7.59
Potassium phosphate monobasic (g)	0.7
N-[Tris (hydroxymethyl) methyl]-2 (g)	2.7
Sodium acetate trihydrate (g)	3.1
Purified water (mL)	100
pH	7.1
Osmolality (mOsm/kg)	310

### 2.4. Motility parameters

Sperm motility and velocity parameters were determined using a computer-assisted sperm analyzer (CASA (SCA, Version 5.1; Microptic, Barcelona, Spain) [18]. To perform this, each sample was adjusted to 10 × 10^6^ sperm/mL and loaded in a pre-warmed chamber slide (37°C, Leja 4; Leja Products, Luzernestraat B.V., Holland). At least five fields including a minimum of 200 sperm, were assessed by CASA. Sperm total motility (TM, %), progressive motility (PM, %), average path velocity (VAP, μm/s), straight linear velocity (VSL, μm/s), curvilinear velocity (VCL, μm/s), and amplitude of lateral head displacement (ALH, μm) were evaluated.

### 2.5. Viability

Sperm viability was evaluated by the eosin-nigrosine method [18]. Five μl of sperm and 10 μl eosin-nigrosine stains were spread on a slide. To detect sperm viability, 200 sperm were assessed under a bright-field microscope (Labomed LX400; Labomed Inc., Culver City, CA, USA) at ×400.

### 2.6. Membrane integrity

Evaluating sperm membrane functionality was performed by Hypoosmotic swelling test (HOST) [18]. The test was performed by adding 10 μL of diluted semen into eppendorf tubes containing 100 mL hypoosmotic solution (1.9 mM sodium citrate and 5 mM fructose, 100 mOsm/kg). After incubation at 37°C for 30 min, 10 μL of the sample was loaded on a microscope slide, and 200 sperm instantly was calculated under phase-contrast microscope (Labomed LX400; Labomed Inc., Culver City, CA, USA) at ×400 to detect sperm membrane functionality.

### 2.7. Morphology

For the assessment of morphology after thawing, 10 μL of sperm was pipetted into tubes including 1 ml of Hancock solution [18] (150 ml sodium saline solution, 150 ml PBS buffer solution and 62.5 ml formalin (37%)). To detect sperm total abnormality, about 200 sperm were counted by phase-contrast microscope (Labomed LX400; Labomed Inc., Culver City, CA, USA) at×1000.

### 2.8. Malondialdehyde (MDA) levels

MDA level was assessed by thiobarbituric acid reaction [19]. In brief, 1 mL of sperm sample was mixed with 1 ml of cold trichloroacetic acid (20%) to precipitate protein. Subsequently, the samples were centrifuged (963×g for 15 min), and 1 ml of the supernatant was incubated with tubes containing 1 ml of thiobarbituric acid (0.67%) in a boiling water bath at 100°C for 10 min. After cooling, the absorbance was assessed by spectrophotometer (T80 UV/VIS PG Instruments Ltd, UK) at 532 nm.

### 2.9. TAC, GPx and SOD assessment

The antioxidant system was examined by the assessment of GPx, TAC, and SOD levels [18]. These variables were assessed spectrophotometrically by Randox™ kits (RANDOX Laboratories Ltd.) and an Olympus AU 400 automatic biochemistry analyzer (Olympus, Tokyo, Japan).

### 2.10. Flow cytometry

Mitochondria activity and apoptosis status were analyzed by FACSCalibur flow cytometer (Becton Dickinson System, San Jose, CA, USA) [18]. The excitation wavelength was 488 nm supplied by an argon laser. The sperm population was gated using forward and side scatter. The volumes of green (Annexin-V and Rhodamine-123) and red fluorescence (PI) were detected respectively with a FL1 photodetector (530 nm) and FL3 photodetector (610 nm). Next, 10×10^3^ events were examined for each assay.

#### 2.10.1. Apoptosis status

**Fig 1:**
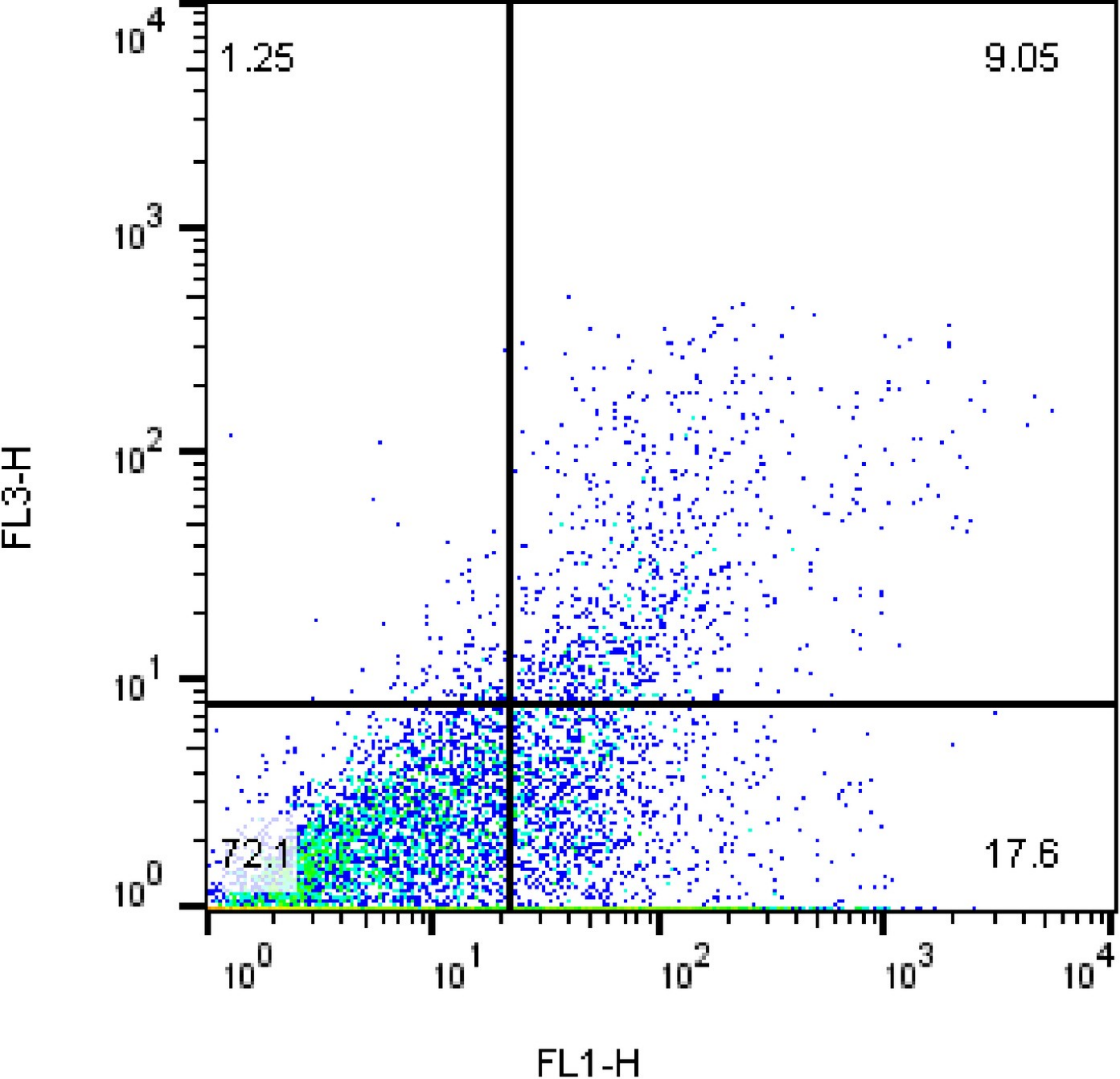
Annexin V (FL1-H) and propidium iodide (FL3-H) staining was used to determine the different cell populations. In each panel, the lower left quadrant shows Annexin-PI- cells (Viable spermatozoa), the lower right shows Annexin+PI- cells (early stage of apoptosis) and upper left and upper right show stained with PI (PI+) (dead spermatozoa).

For detection of sperm apoptosis status [18], the sperm samples were washed in calcium buffer and then, 10 μL Annexin V FITC (AV) (0.01 mg/mL) was added to 100 μL sperm suspension. Following incubating for a minimum of 20 min at room temperature, 10 μL of propidium iodide (PI) (1 mg/mL) was added to the sperm suspension, then incubated for 10 min before flow cytometry evaluation. Following flow cytometry, sperm subpopulations process were classified into three various groups including (1) viable sperm (AV-/PI-); (2) early apoptotic sperm (AV+/PI-); (3) and dead spermatozoa, stained with PI (PI+) (**Fig 1**).

#### 2.10.2. Mitochondrial activity

**Fig 2:**
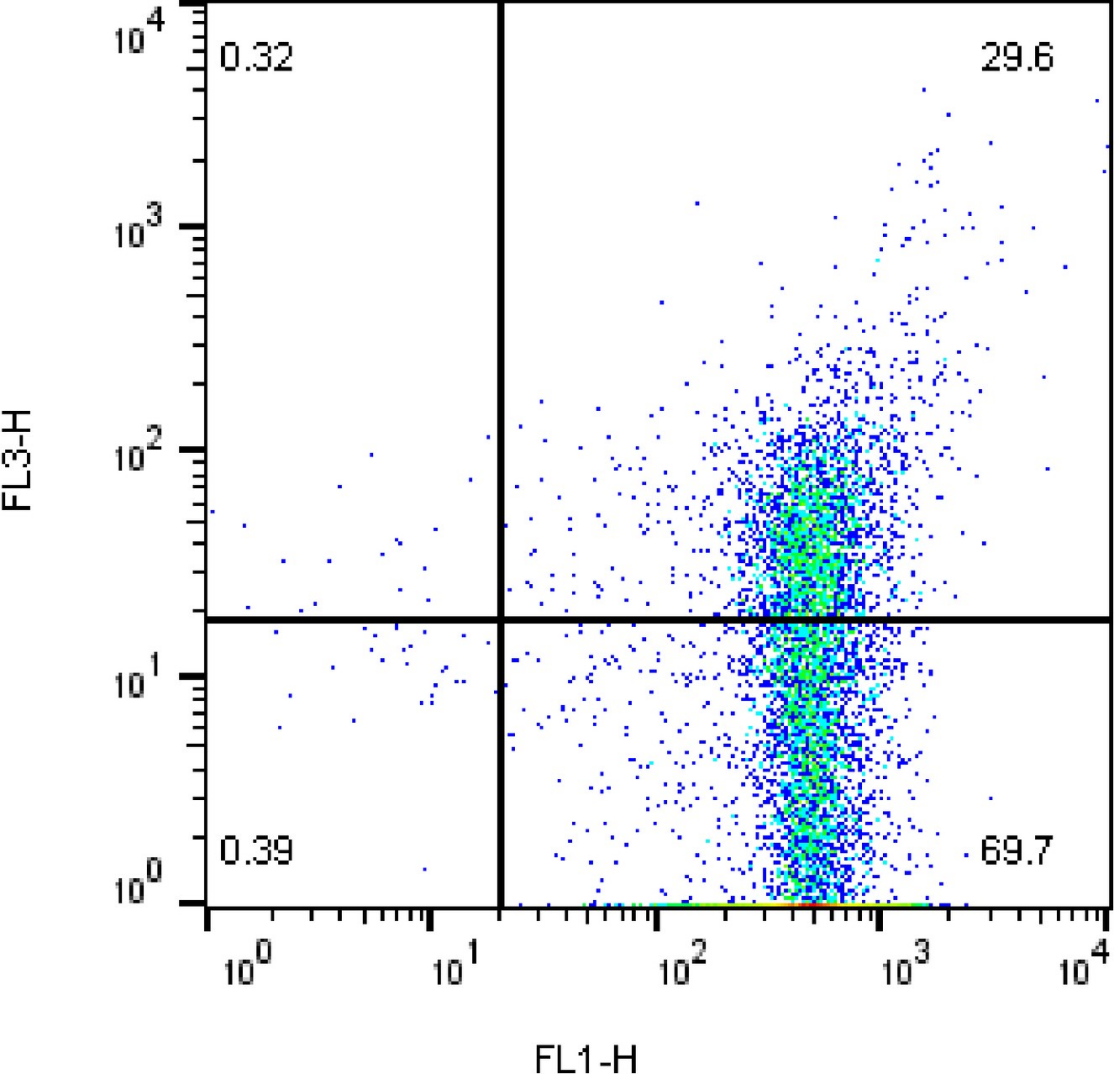
Flow cytometric detection of rooster sperm stained with Rhodamine123 (FL1-H) and PI (FL3-H) after freeze-thaw process. R123+PI- quadrant contains spermatozoa with active mitochondria; R123+ PI+ quadrant contains dead spermatozoa.

Mitochondrial activity was assessed by Rhodamine 123 (R123) (0.01 mg/ml) and PI (1 mg/mL) staining [18]. In brief, 5 microliters of R123 solution (0.01 mg/ml) and PI were added to 250 μl of diluted semen sample and then incubated in a dark place for 20 min at 37°C. Finally, the percentage of active mitochondria (positive signal for R123 and negative signal for PI) was studied by flow cytometer (**Fig 2**).

### 2.11. RNA extraction and real-time polymerase chain reaction

Total RNA was extracted from sperm samples using Trizol reagent (Invitrogen, Carlsbad, CA, USA) by the method provided by the manufacturer and quantified using ND-1000 spectrophotometer (NanoDrop Technologies, Wilmington, DE, USA). RNA was transcribed into complementary DNA by the reverse transcription reagent kit (REVERTA-L RT reagents kit; code: K3-4-100-CE) and a thermal cycler according to manufacturer’s instructions. The RT reaction was conducted in 20 μL of the reaction mixture at 37°C for 30 minutes and then the samples were stored at ≤ –20°C.

Primers were designed using Primer3Plus online software on the basis of the GenBank sequence of target genes and are presented in Table 2. The specificity of the primers was checked by BLAST analysis of the National Center for Biotechnology information’s database. In the meantime, GAPDH was amplified as an endogenous control gene.

**Table 2:** Primer sequences used for quantitative real-time polymerase chain reaction.

Gene	Primer sequence (5'–3')	Product size (bp)	Accession no.
GAPDH	F: ATCACAGCCACACAGAAGACG	120	NM_204305.1
	R: GACTTTCCCCACAGCCTTAGC		
CASPASE 3	F: AACCAGCCTTTTCAGAGGTGAC	119	NM_204725.1
	R: CTGGTCCACTGTCTGCTTCAATA		
BCL-2	F: AACATTGCCACCTGGATGAC	118	NM_205339.2
	R: CGAACAAAGGCCTCATACTGT		

All polymerase chain reactions (PCRs) were carried out in ABI StepOnePlus Real-Time PCR Systems (Applied Biosystems, USA) using the RealQ Plus 2x Master Mix Green Kit (Ampliqon, code: A325402) following manufacturer’s instructions. As a whole, the reaction was performed at 95°C for 15 min, followed by 40 cycles of denaturing, annealing and elongating (95°C for 15 seconds, 61°C for 20 seconds and 72°C for 30 seconds, respectively). The dissociation curves of PCR products were achieved by a following cycle of 95°C for 15 seconds, 60°C for 1 min and 95°C for 15 seconds, and the reaction specificity was defined when there was only one specific peak in the dissociation curve. The R^2^ values for all standard curves generated ranged 0.999, and PCR efficiencies was ≥95%. The quantitative PCR data were analyzed using the 2^-ΔΔCt^ method [20]. The mRNA expressions of pro-apoptotic (CASPASE 3) and anti-apoptotic (Bcl-2) genes were assessed after freeze-thawing.

### 2.12. Fertility test

Reproductive performance of post-thaw sperm was assessed by artificial insemination [3]. Thirty Ross broiler breeder hens (28 weeks old) were caged (n = 10 hens/each group) and inseminated with frozen-thaw sperm in three experimental groups that were selected according to the results of in-vitro sperm assessments. Then the assessment of fertility in naringenin at 100 μM (N100), crocin at 1 mM (C1) and control groups was performed. Artificial insemination was performed at 15 pm on certain days (twice a week for approximately two weeks) with insemination of 100 × 10^6^ (sperm concentration was determined using a hemocytometer (HBG, Berlin, Germany)) sperm per hen obtained from each treatment. Eggs were collected for five days after the last artificial insemination. For each group, 50 eggs in 2-weeks were randomly selected for incubation. The eggs were incubated in a commercial incubator.

The fertility of the eggs were evaluated on seventh day of incubation by candling. Hatched eggs were counted after 21 days of incubation, and the hatching ratio was calculated based on the number of fertilized eggs.

### 2.13. Statistical analysis

The experiment was performed in five replicates. All data were assessed for normal distribution by PROC UNIVARIATE and the Shapiro–Wilk test. Arc sine (for percentage data) transformation of data was used when appropriate. Data obtained from post-thawing quality were analyzed by PROC GLM SAS 9.1 (version 9.1, 2002, USA). The model used in the present study was: Y_ij_ = μ + T_i_ + e_ij_ which Y_ij_ stands for observed depended variables including sperm parameters, μ is the mean of the population, T_i_ is the effect of treatment, e_ij_ is the random residual error. The effects of the supplemented antioxidants on fertility and hatchability were analyzed using GENMOD procedure. The results are expressed as the mean±SEM. The Tukey's test was performed to compare treatments. The significance level was p < 0.05.

## 3. Results

Motility and velocity parameters of frozen-thawed rooster sperm supplemented with different levels of crocin and naringenin are presented in Table 3. C1 and N100 resulted in higher (P < 0.05) sperm total motility (TM) (74.43±0.79 and 71.21±1.47, respectively) compared to the control group (61.06±1.64). The highest values for sperm progressive motility (PM) were achieved in the C1 and N100 groups (30.88±0.74 and 28.46±2.38, respectively) when compared with control group (22.03±1.27). The analysis did not reveal any significant differences among different concentrations of crocin and naringenin on the VCL, VAP, VSL, ALH, LIN, BCF and STR parameters.

**Table 3:** Effect of different levels of crocin and naringenin on motility parameters of rooster thawed semen, analyzed by CASA (*n* = 5).

Antioxidant	TM (%)	PM (%)	VSL (μm/s)	VAP (μm/s)	VCL (μm/s)	LIN (%)	STR (%)	ALH (μm)	BCF (Hz)
Control	61.06±1.64^c^	22.03±1.27^b^	16.37±1.35	29.94±1.31	52.47±4.70	32.88±4.35	54.65±3.51	5.25±0.22	15.33±0.63
C0.5	65.19±1.89^bc^	26.66±2.40^ab^	17.42±2.16	31.74±1.86	56.22±1.53	31.32±3.31	54.80±8.60	4.94±0.28	15.72±1.05
C1	74.43±0.79^a^	30.88±0.74^a^	18.78±0.93	33.13±2.49	57.72±1.01	33.03±1.87	57.88±2.85	4.58±0.10	16.10±0.81
C1.5	61.34±1.86^c^	25.10±0.36^ab^	16.76±1.59	30.42±1.43	54.08±0.86	31.28±3.82	55.86±7.07	4.90±0.23	15.17±1.39
N50	64.13±1.33^bc^	24.58±1.24^ab^	17.30±1.21	31.24±0.72	53.23±1.81	32.14±2.91	56.51±3.32	5.28±0.36	15.59±2.25
N100	71.21±1.47^ab^	28.46±2.38^a^	18.68±1.45	32.01±2.46	57.13±2.32	33.02±3.37	59.25±4.83	4.78±0.25	16.06±2.75
N150	60.94±1.84^c^	21.61±1.60^b^	16.54±1.19	30.33±1.20	51.08±1.23	31.26±2.87	55.59±5.23	5.04±0.07	15.49±0.88

TM: Total motility (%); PM: Progressive motility (%); VSL: straight-line velocity (μm/s); VAP: Average path velocity (μm/s); VCL: curvilinear velocity (μm/s); LIN: Linearity (%); STR: Straightness (%); ALH: Mean amplitude of the lateral head displacement (μm); BCF: Mean of the beat cross frequency (Hz). Different superscripts within the same column indicate significant differences among groups (p < 0.05).

Beltsville extender without antioxidant (control), C0.5 (Beltsville extender with 0.5 mM crocin), C1 (Beltsville extender with 1 mM crocin), C1.5 (Beltsville extender with 1.5 mM crocin), N50 (Beltsville extender with 50 μM naringenin), N100 (Beltsville extender with 100 μM naringenin), N150 (Beltsville extender with 150 μM naringenin).

**Fig 3:**
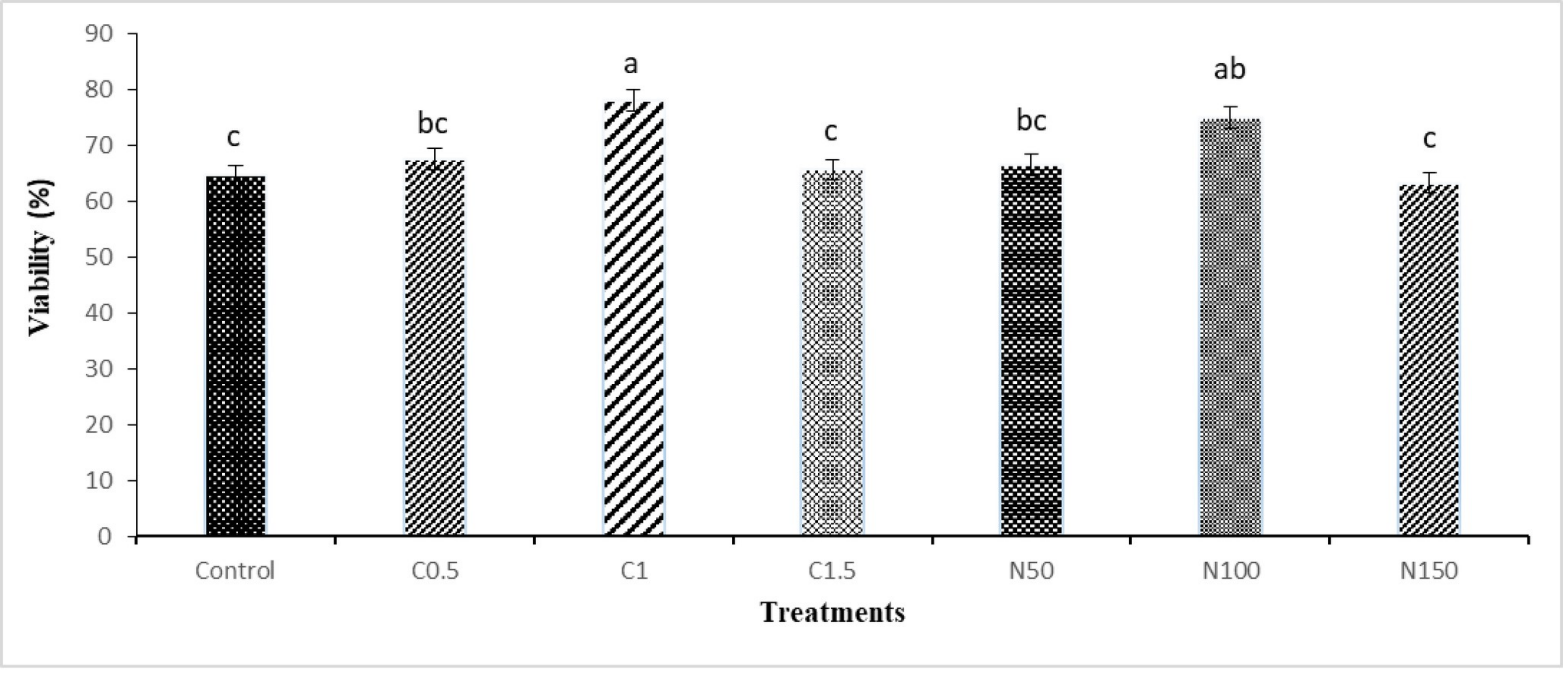
Effect of crocin and naringenin supplementation in cryopreservation medium on post thawed viability (%, eosin-nigrosine method) of rooster sperm. Beltsville extender without antioxidant (control), C0.5 (Beltsville extender with 0.5 mM crocin), C1 (Beltsville extender with 1 mM crocin), C1.5 (Beltsville extender with 1.5 mM crocin), N50 (Beltsville extender with 50 μM naringenin), N100 (Beltsville extender with 100 μM naringenin), N150 (Beltsville extender with 150 μM naringenin).

The results of viability (eosin-nigrosine method) are presented in **Fig 3**. The results showed that viability (eosin-nigrosine method) of sperm in C1 and N100 groups were improved compared with control.

**Fig 4:**
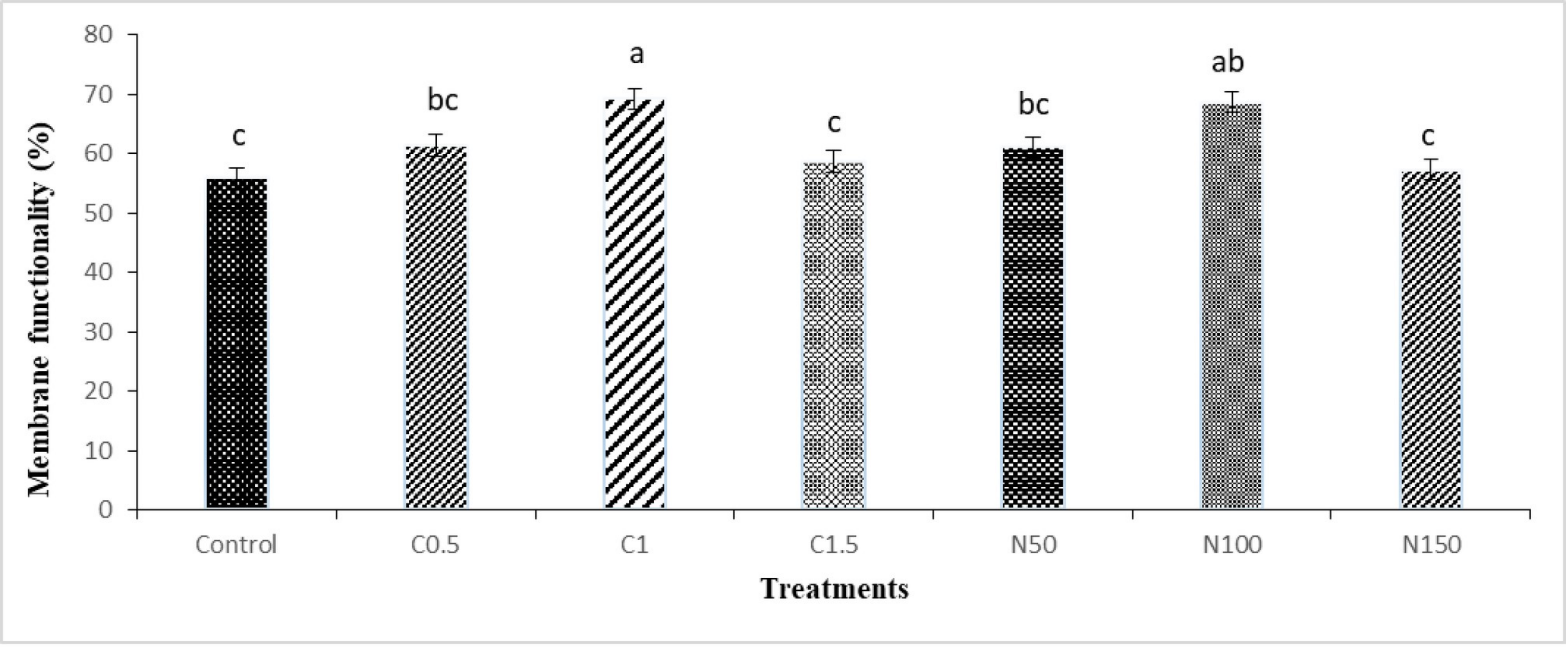
Effect of crocin and naringenin supplementation in cryopreservation medium on post-thawed membrane functionality (%, HOS-test) of rooster sperm. Beltsville extender without antioxidant (control), C0.5 (Beltsville extender with 0.5 mM crocin), C1 (Beltsville extender with 1 mM crocin), C1.5 (Beltsville extender with 1.5 mM crocin), N50 (Beltsville extender with 50 μM naringenin), N100 (Beltsville extender with 100 μM naringenin), N150 (Beltsville extender with 150 μM naringenin).

The results of membrane functionality showed that plasma membrane functionality in C1 and N100 groups were significantly higher compared to the control group **(Fig 4)**.

**Fig 5:**
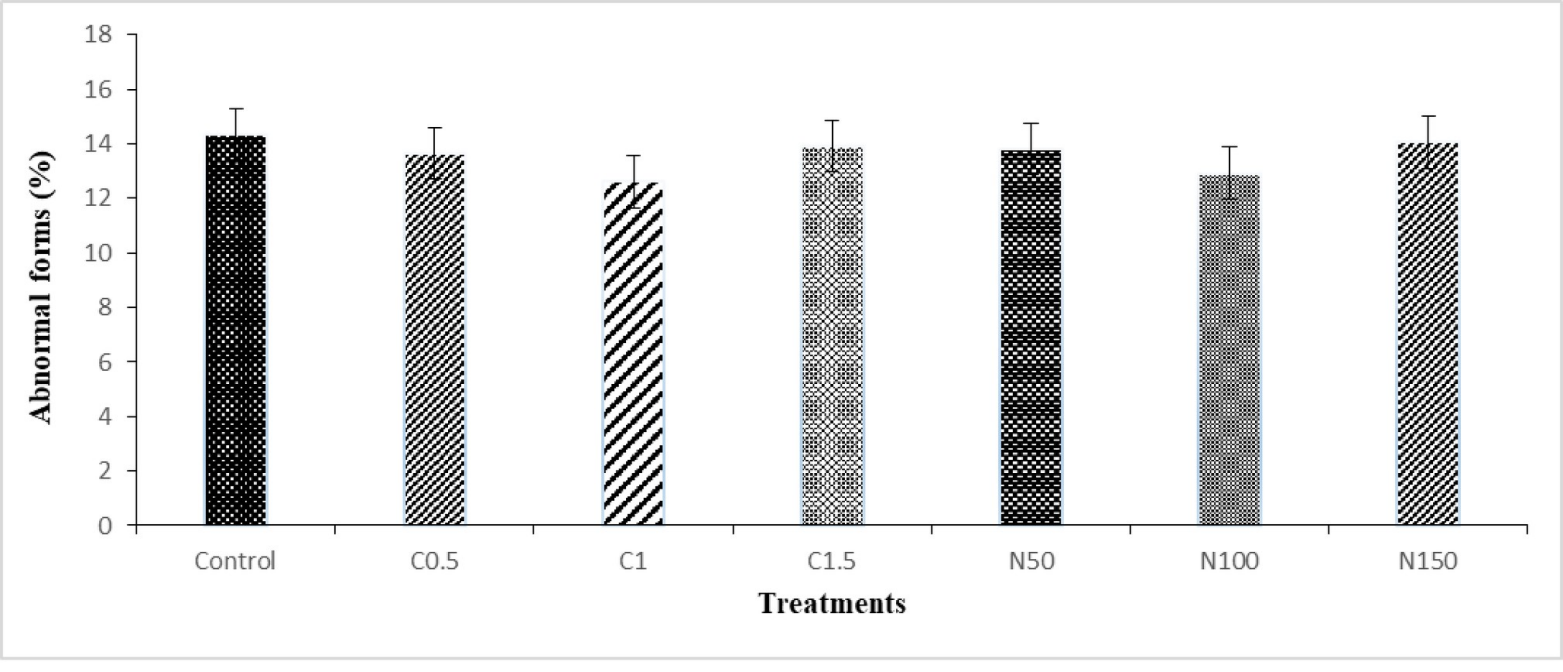
Effect of crocin and naringenin supplementation in cryopreservation medium on post-thawed abnormal forms (%, Hancock solution) of rooster sperm. Beltsville extender without antioxidant (control), C0.5 (Beltsville extender with 0.5 mM crocin), C1 (Beltsville extender with 1 mM crocin), C1.5 (Beltsville extender with 1.5 mM crocin), N50 (Beltsville extender with 50 μM naringenin), N100 (Beltsville extender with 100 μM naringenin), N150 (Beltsville extender with 150 μM naringenin).

The results showed that different levels of crocin and naringenin did not affect the abnormal forms after freeze-thawing (**Fig 5**).

**Table** 4 reports the data on the effects of different levels of crocin and naringenin on the oxidative parameters status of rooster sperm following freeze-thawing. The highest values for TAC activity were achieved in the C1 and N100 groups (1.85±0.18 and 1.88±0.05, respectively) compared to control group (1.13±0.18). Also, malondialdehyde was significantly (P < 0.05) lower in C1 and N100 groups (1.83±0.08 and 1.90±0.09, respectively) when compared to the control group (4.26±0.18). The analysis did not reveal any significant differences for SOD and GPx parameters.

**Table 4:** Effect of different levels of crocin and naringenin on malondialdehyde concentration (MDA), glutathione peroxidase (GPx) and superoxide dismutase (SOD) activities and total antioxidant capacity (TAC) of rooster thawed semen (n = 5).

Antioxidant	MDA (nmol/mL)	GPx (U/mg protein)	SOD (U/mg)	TAC (mmol/l)
Control	4.26±0.18^a^	54.00±1.71	107.70±6.30	1.13±0.18^bc^
C0.5	2.91±0.17^c^	60.70±3.07	118.25±2.41	1.70±0.04^ab^
C1	1.83±0.08^d^	63.20±1.88	124.65±4.34	1.85±0.18^a^
C1.5	3.81±0.12^ab^	55.20±1.40	109.38±5.92	1.26±0.12^bc^
N50	3.12±0.27^bc^	57.85±1.23	117.87±9.98	1.66±0.03^abc^
N100	1.90±0.09^d^	62.71±1.71	123.92±2.74	1.88±0.05^a^
N150	4.01±0.19^a^	53.61±1.85	108.37±4.29	1.11±0.07^c^

Different superscripts within the same column indicate significant differences among groups (P < 0.05). Beltsville extender without antioxidant (control), C0.5 (Beltsville extender with 0.5 mM crocin), C1 (Beltsville extender with 1 mM crocin), C1.5 (Beltsville extender with 1.5 mM crocin), N50 (Beltsville extender with 50 μM naringenin), N100 (Beltsville extender with 100 μM naringenin), N150 (Beltsville extender with 150 μM naringenin).

**Table** 5 depicts the data on apoptosis status analysis. The most remarkable result is that the percentage of viable (AnnexinV-/PI-) sperm was higher in 1 mM crocin and 100 μM naringenin (71.46±2.00 and 70.86±1.23, respectively) in comparison with control group (56.95±0.93). Early apoptosis spermatozoa were significantly reduced in the C1 and N100 groups (15.30±0.32 and 15.40±2.10, respectively) when compared to the control group (25.28±1.04).

**Table 5:** Effect of different levels of crocin and naringenin on viable, apoptotic and dead sperm in rooster thawed semen, as assessed by flow cytometry (*n* = 5).

Antioxidant	Viable (%)	Early apoptosis (%)	Dead (%)
Control	56.95±0.93^b^	25.28±1.04^a^	17.76±1.63
C0.5	60.88±1.27^b^	21.88±2.41^ab^	17.23±1.77
C1	71.46±2.00^a^	15.30±0.32^b^	13.23±1.82
C1.5	57.31±0.84^b^	24.47±1.34^a^	18.20±1.11
N50	59.73±1.04^b^	22.81±1.17^ab^	17.45±1.06
N100	70.86±1.23^a^	15.40±2.10^b^	13.72±1.88
N150	57.24±1.06^b^	24.78±1.93^a^	17.97±2.13

Viable (%, AnnexinV-/PI-), early apoptosis (%, AnnexinV+/PI-) and dead (%, PI+) parameters were analyzed. Different superscripts within the same column indicate significant differences among groups (p < 0.05). Beltsville extender without antioxidant (control), C0.5 (Beltsville extender with 0.5 mM crocin), C1 (Beltsville extender with 1 mM crocin), C1.5 (Beltsville extender with 1.5 mM crocin), N50 (Beltsville extender with 50 μM naringenin), N100 (Beltsville extender with 100 μM naringenin), N150 (Beltsville extender with 150 μM naringenin).

**Fig 6:**
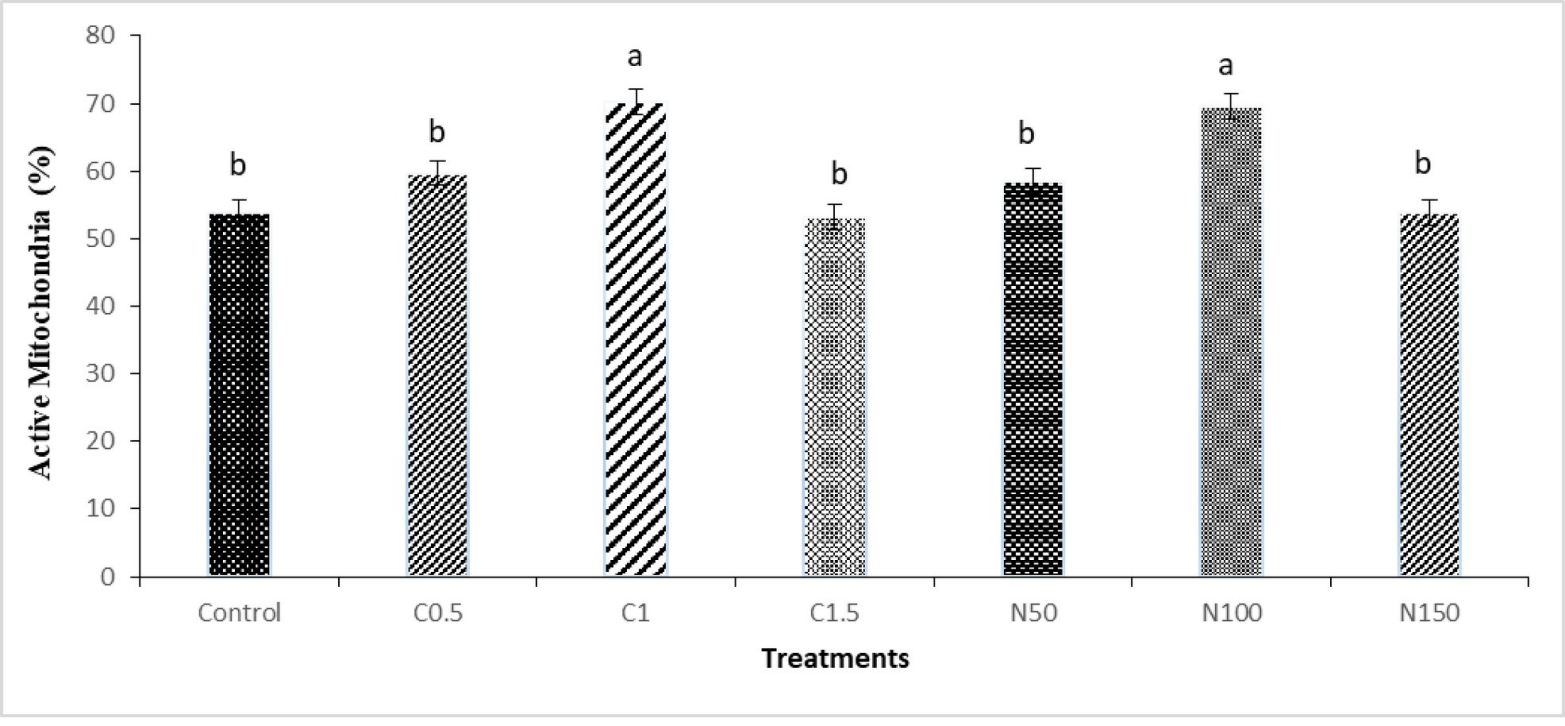
Effect of crocin and naringenin supplementation in cryopreservation medium on post-thawed active mitochondria (%, R123+/PI-) of rooster sperm. Beltsville extender without antioxidant (control), C0.5 (Beltsville extender with 0.5 mM crocin), C1 (Beltsville extender with 1 mM crocin), C1.5 (Beltsville extender with 1.5 mM crocin), N50 (Beltsville extender with 50 μM naringenin), N100 (Beltsville extender with 100 μM naringenin), N150 (Beltsville extender with 150 μM naringenin).

The results on mitochondrial activity revealed that the percentage of active mitochondria was higher in the C1 and N100 groups when compared to the control group (**Fig 6**).

**Fig 7:**
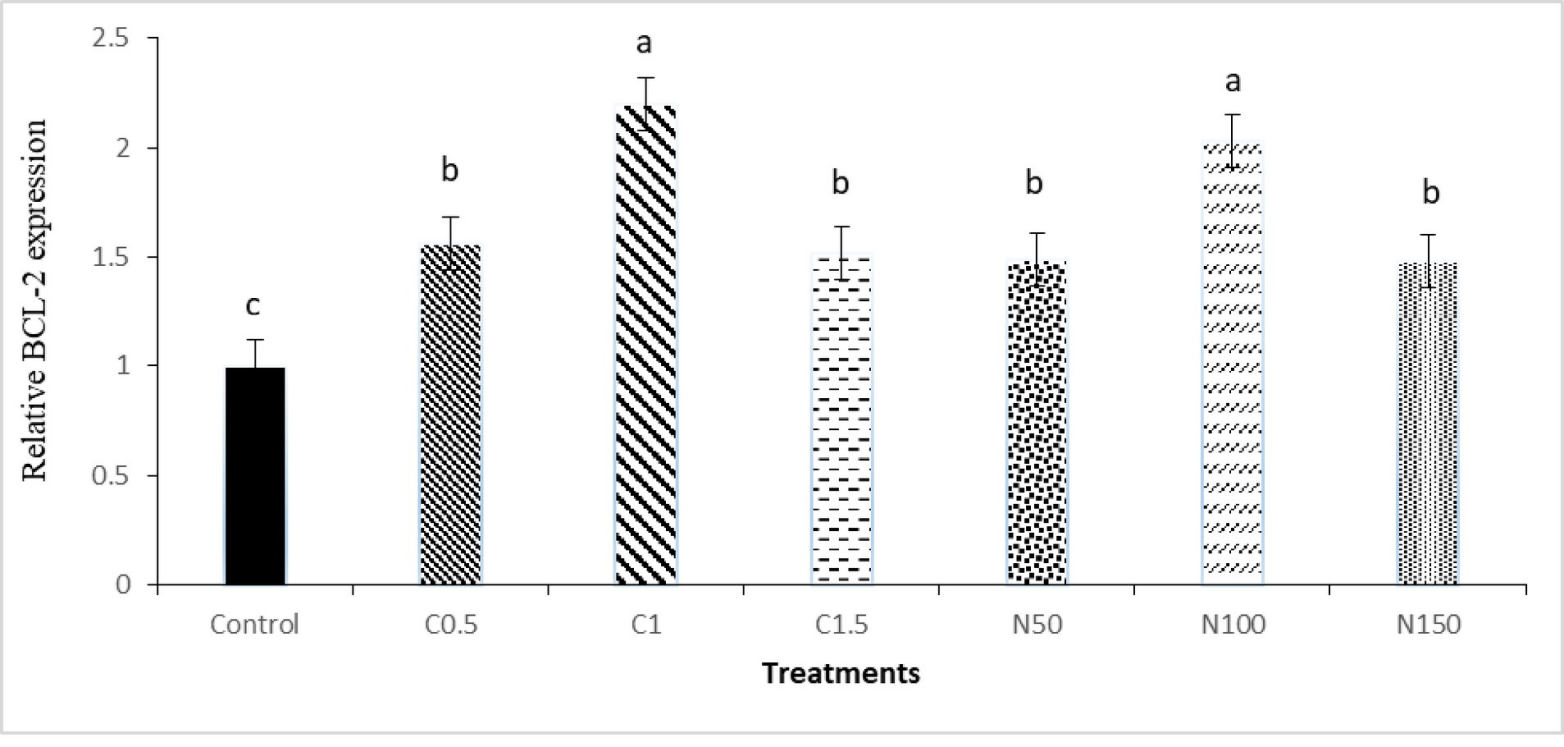
Relative mRNA expression of BCL-2 gene in the rooster sperm cryopreservation supplemented with crocin and naringenin. Beltsville extender without antioxidant (control), C0.5 (Beltsville extender with 0.5 mM crocin), C1 (Beltsville extender with 1 mM crocin), C1.5 (Beltsville extender with 1.5 mM crocin), N50 (Beltsville extender with 50 μM naringenin), N100 (Beltsville extender with 100 μM naringenin), N150 (Beltsville extender with 150 μM naringenin).

**Fig 8:**
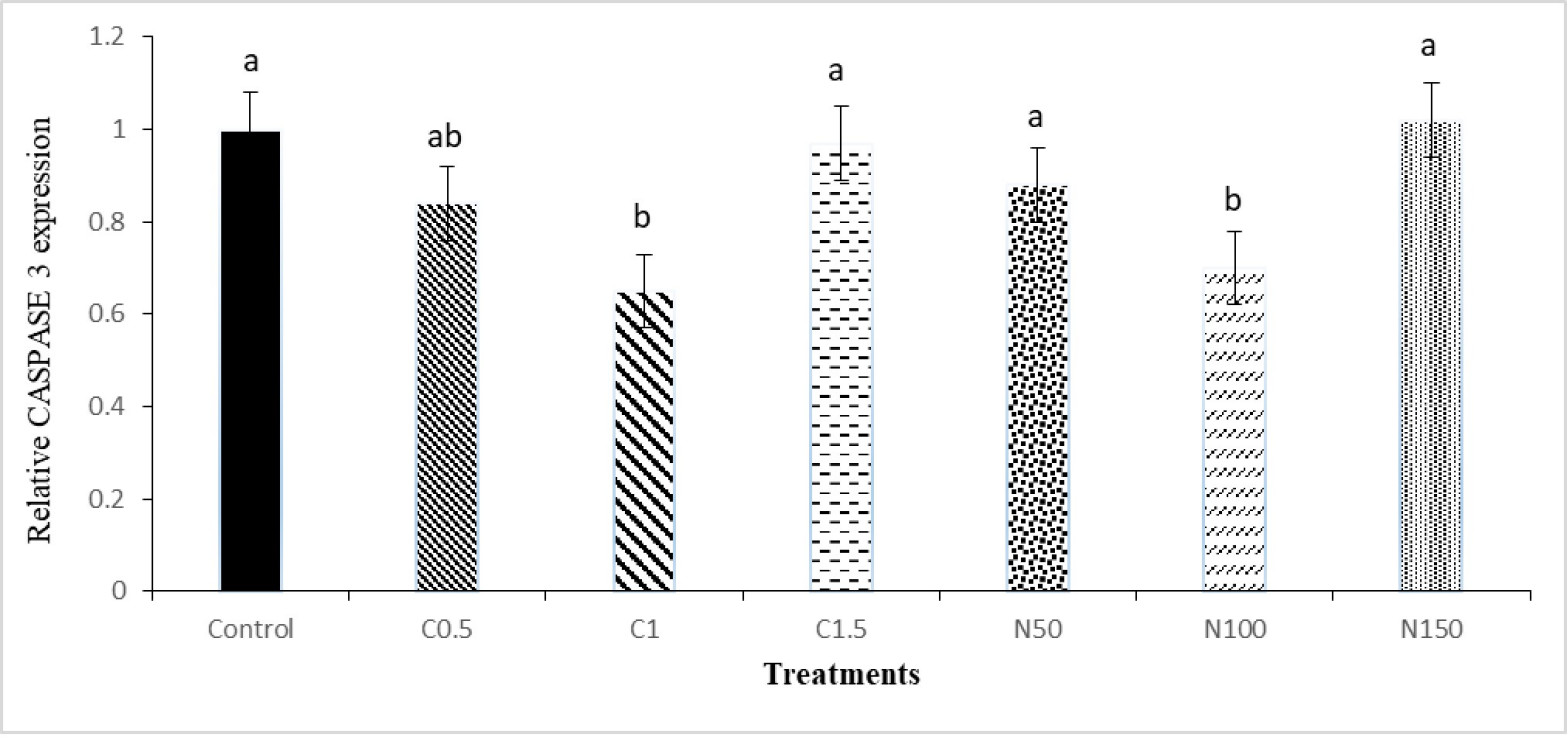
Relative mRNA expression of CASPASE 3 gene in the rooster sperm cryopreservation supplemented with crocin and naringenin. Beltsville extender without antioxidant (control), C0.5 (Beltsville extender with 0.5 mM crocin), C1 (Beltsville extender with 1 mM crocin), C1.5 (Beltsville extender with 1.5 mM crocin), N50 (Beltsville extender with 50 μM naringenin), N100 (Beltsville extender with 100 μM naringenin), N150 (Beltsville extender with 150 μM naringenin).

The results of mRNA expressions of BCL-2 and CASPASE 3 are shown in Figs 7 and 8. The mRNA expressions of BCL-2 in the C1 and N100 groups were significantly higher than the other treatments. The expression of CASPASE 3 was significantly reduced in C1 and N100 group (P < 0.05) compared to control group.

The results of fertility test are presented in **Table** 6. The findings of the fertility test revealed significantly higher (P < 0.05) percentages of fertile eggs, hatched eggs and hatching rate in C1 (52, 38 and 73.08, respectively) and N100 (54, 40 and 74.07, respectively compared to the control group (38, 18 and 47.37, respectively).

**Table 6:** Effect of crocin and naringenin on fertility and hatchability rates of rooster semen after freeze-thawing. Each experimental group contained 50 eggs initially. Numbers are absolute counts of eggs, with percentages (ratio respect to the initial egg count) between parentheses, except for the hatched eggs ratio.

Antioxidant	Fertilized eggs	Hatched eggs	Hatched eggs ratio (hatched/fertilized, %)
Control	19 (38)^b^	9 (18)^b^	47.37^b^
Naringenin 100 μM	27 (54)^a^	20 (40)^a^	74.07^a^
Crocin 1 mM	26 (52)^a^	19 (38)^a^	73.08^a^

Different superscripts letters within column are significantly different (P < 0.05).

## 4. Discussion

Studies evaluating the efficacy of antioxidants to prevent sperm damage during cryopreservation usually cause contradictory results. Some experiments have reported protective effects against cryo-related oxidative damages [21]. However, other studies could not show significant effects; some even led to impaired sperm function [7].

Free radicals produce changes of spermatozoa composition by activating intracellular pathways leading to activating processes such as chromatin condensation, motility, capacitation, acrosome reaction, and chemotaxis. Conversely, excessive amounts of ROS have pathological effects on spermatozoa ranging from diminished sperm concentration, decreased motility and reduced fertilization. ROS, therefore act as a double edge sword [22]. Although semen possesses an antioxidant system, their activity is affected by cryopreservation, which increases the intensity of lipid peroxidation. Therefore, natural antioxidants may be insufficient to prevent lipid peroxidation on sperm cells during the freezing–thawing process. Therefore, the addition of antioxidants to the extender may have positive effects.

Some points must be taken into consideration when performing an antioxidant treatment. The most important point is that each kind of ROS is deactivated by a specific antioxidant system [23]; therefore, if antioxidant therapy is chosen at random, this treatment will not be effective because it may not directly decrease oxidative damage [24]. The protective effect of natural antioxidants on oxidative stress in avian species has been considered in various studies. It is demonstrated that oxidative stress can be reduced by adding a variety of antioxidants to poultry sperm [25]. Some properties of crocin and naringenin make them highly effective supplements to be used as additives for rooster sperm cryopreservation extender. The hypothesis in the present study was that crocin and naringenin, as a supplement in freezing extenders, could be effective in eliminating oxidative damage caused by freezing. Interestingly, using high levels of the naringenin at 150 μM (N150) and crocin at 1.5 mM (C1.5) did not yield any positive effects, reverting the quality parameters to values comparable to the control. Antioxidants might have negative effects due to excessive scavenging of free radicals, possibly by altering their physiological levels.

It was observed in our study that adding 1 mM of crocin and 100 μM naringenin during the preparation of the sperm had a beneficial effect on the total and progressive motility of sperm in comparison with the control group, while no significant effect was observed on the other motility parameters. The favorable effect of saffron and its bioactive component, crocin, on some parameters such as motility and viability has been demonstrated in different study [26]. It is demonstrated that in stressful conditions, naringenin can chelate irons and decrease ROS production. Interestingly, it is related to the fact that naringenin has 5-hydroxy and 4-carbonyl groups in the C-ring which play an important role in ROS scavenging [27]. Therefore, adding naringenin to the cryopreservation medium can decrease the stress caused by freezing, consequently increases motility which was observed in our study.

It is shown that crocin can reduce the levels of superoxide anion and hydrogen peroxide. The supplementation of crocin in the cryopreservation extender showed to be advantageous for the viable sperm (AnnexinV-/PI-) at the C1 group. Carotenoids show stabilizing effect on sperm conservation by interaction with the superoxide anion [28]. Furthermore, crocin enhances the activity of particular intracellular detoxifying enzymes or affects the fluidity of the membrane, which influences its permeability to oxygen and further molecules [29].

Our previous studies have adopted an approach in the study of the relation between sperm variables and MDA levels [30]. The correlation between MDA content of the sperm and the fertilization capacity is worth mentioning [31]. Malondialdehyde levels in semen are inversely related to the sperm function [24]. These data were again confirmed in the present investigation, in which the MDA level was evaluated because it is known as a gold marker for oxidative stress, a phenomenon extremely associated with the antioxidant system. In line with our study, Sapanidou et al. [11], showed that MDA production decreased while supplementing 1 mM crocin in sperm.

According to our results, naringenin 100 μM reduced the MDA level. A satisfactory explanation for this, may be related to its structure-activity. Naringenin can give hydrogen to ROS that allows the acquisition of stable composition and eliminates these free radicals. Another interesting reason is the existence of phenolic rings in naringenin which act as electron barriers to remove superoxide anions known as free radicals [32].

In the light of the data, it is clearly essential to comprehend what cellular factors normally serve as motivators for free radical production by sperm mitochondria. It is demonstrated that the production of mitochondrial ROS raises when the membrane potential is damaged [33]. Carotenoids have a recognized protective effect in the mitochondria and crocin itself has been reported as a mitochondrial protector [34]. Therefore, it was predictable that C1 and N100 increased mitochondrial activity after thawing. The axosoma and dense fibers associated with the central part of the sperm cells are covered by mitochondria, the organs which produce energy from ATP that are involved in sperm motility [35]. It is obvious that cryopreservation results in a reduction in sperm motility, membrane functionality and mitochondrial membrane potential [36]. The unique physiological characteristics of avian spermatozoa such as high sperm concentration, elongated size, less seminal plasma, and cytoplasm showed step-up oxidative intensiveness [37]. It is shown that during freeze-thawing of semen samples, damaged and dead sperm cells normally are higher in avian than in mammal spermatozoa, moreover, they increase the level of ROS molecules [38]. Because avian sperm membrane involves a rich amount of polyunsaturated fatty acid, they are prone to lipid peroxidation as exposed to ROS during the process of freezing and thawing [39]. Reports from researchers reveal that the decrease in sperm quality parameters such as sperm motility, is associated with decrease in antioxidant potential of frozen-thawed semen [25]. An obvious correlation has been confirmed between sperm motility and mitochondrial activity [3]. Therefore, it was logical in the present study that supplementation of sperm extender with crocin 1mM and 100 μM naringenin before cryopreservation increased membrane functionality and mitochondrial activity leading to improvement of sperm motility.

Mitochondrial dysfunction is shown to be a critical modulator of ROS production and consequently the onset of apoptosis. An interesting result was found for crocin 1 mM and 100 μM naringenin in reducing early apoptosis. This is in complete agreement with Sapanidou et al. [11], who reported that PS externalization decreased in the group containing 1 mM crocin. Our results do not support the observations reported by Mata-Campuzano et al. [40], who noted that crocin did not affect apoptotic ratio in ram sperm following cryopreservation. It is indicated that various apoptogenic proteins containing Cyt-c, AIF and Endo-G are released through pores generated by the mitochondrial membrane potential and consequently inhibiting the release of different types of apoptogenic factors from mitochondria. Thereby, the expressions of caspase-3 and bcl-2 which were regulated in sperm cells owing to the release of apoptogenic factors from mitochondrial pores were inhibited in naringenin 100 μM and crocin 1 mM. As explained above, naringenin is effective in conserving the mitochondrial membrane by preventing the excessive production of ROS, consequently, inhibiting the release of several apoptogenic factors from the mitochondria [41]. Also it is shown that naringenin restricts translocation of AIF and Endo-G to the nucleus by restoring mitochondrial membrane potential that prevents DNA damage and, finally inhibits cell damage [42]. It is an appreciable reason for preventing apoptosis by naringenin after freeze-thawing.

The results of this study show that the increase in fertility using thawed sperm stored in C1 and N100 was consistent with the other sperm functional parameters. The freezing and thawing process dramatically reduces the fertilization capacity of the rooster sperm. Likewise, a relatively large number of live sperm is required inside the sperm storage tubes (SST) to determine fertilization after inseminations [43]. C1 and N100 treatments improved sperm motility and plasma membrane functionality, consequently increased the population of useful spermatozoa in the SST. Successful fertilization is associated with the optimal sperm motility, membrane functionality and viability of sperm [44]. It has been shown that the number of sperm penetration in inner perivitelline layer is positively correlated with fertility and sperm storage in the SST [45]. So, the strategies that enhance the sperm viability and motility will ensure sperm journey in the hen reproductive tract to reach SST and then the fertilization position. Also, improving sperm antioxidant system and mitochondria activity will increase sperm function during passage in the reproductive tract [46]. Therefore, it appears that higher sperm quality in groups of C1 and N100 showed higher hatching rate among treatment groups by preserving more alive sperm in SST and influencing fertility in the current study.

The present study showed that 1 mM crocin and 100 μM naringenin could beneficially affect sperm quality in Ross 308 breeder roosters. Particularly, 1 mM crocin and 100 μM naringenin could protect the sperm by reducing the pro-apoptotic (CASPASE 3) and increasing anti-apoptotic (Bcl-2) apoptosis genes. Also, enrichment of semen extender with 1 mM crocin and 100 μM naringenin improved the fertilizing capacity of rooster sperm.

## Supporting information

S1 Data (XLS)

S1 File (DOC)
