# Supplementary material for: Effect of crocin and naringenin supplementation in cryopreservation medium on post-thaw rooster sperm quality and expression of apoptosis associated genes
Source: PLoS One. 2020 Oct 29;15(10):e0241105. doi: 10.1371/journal.pone.0241105 (PMC7595379; doi:10.1371/journal.pone.0241105)
Supplement: S1 File — (DOC) [file pone.0241105.s002.doc]

- CASPASE 3 was significantly reduced in C1 and N100 group.
- Higher percentage of fertility and hatching rate were observed in C1 and N100.
- Sperm mitochondrial activity and membrane functionality were higher in C1 and N100.
- Apoptotic sperm were significantly reduced in the C1 and N100.
